# Investigation on the efficacy of a tissue equivalent material bolus for BNCT

**DOI:** 10.1002/acm2.70287

**Published:** 2025-10-10

**Authors:** Akinori Sasaki, Naonori Hu, Ryo Kakino, Mai Nojiri, Keiji Nihei, Teruhito Aihara, Satoshi Takeno, Yuki Yoshino, Hiroki Tanaka, Koji Ono

**Affiliations:** ^1^ Osaka Medical and Pharmaceutical University, Kansai BNCT Medical Center Osaka Japan; ^2^ Kyoto University, Institute for Integrated Radiation and Nuclear Science Kyoto Japan; ^3^ Department of Radiation Oncology Osaka Medical and Pharmaceutical University Hospital Osaka Japan; ^4^ Department of Otorhinolaryngology Head and Neck Surgery Osaka Medical and Pharmaceutical University Hospital Osaka Japan; ^5^ Department of Radiology Kyoto Prefectural University of Medicine Kyoto Japan

**Keywords:** BNCT, bolus, neutron activation

## Abstract

**Background:**

In radiation therapy, the use of bolus is an effective technique for improving the surface dose. This irradiation technique is also used in BNCT. However, since BNCT uses neutron irradiation, it is important to evaluate both the neutron moderation characteristic and the radioactivation of the bolus.

**Purpose:**

This study aimed to evaluate the tissue equivalence and activation of commercially available boluses for use in BNCT.

**Methods:**

Two types of commercially available boluses were evaluated. The boluses were placed on a water phantom and irradiated using the NeuCure BNCT system. Firstly, the tissue equivalency of the boluses was evaluated by comparing the experimentally measured thermal neutron flux and gamma‐ray distribution within the phantom, and the results were compared with simulation results. Secondly, the neutron activation of the boluses was assessed using an ionization chamber survey meter and an HP‐Ge semiconductor detector to identify the produced radionuclides.

**Results:**

The thermal neutron flux and gamma ray distribution in the water phantom agreed well between the measured and simulated results. The study revealed that both boluses became radioactive after neutron irradiation, primarily due to the production of radionuclides such as ^24^Na and ^38^Cl.

**Conclusions:**

While boluses are effective in improving surface dose in BNCT, their use also introduces the risk of patient exposure to radiation from radioactivated bolus materials. Therefore, careful selection of bolus materials with minimal radioactivation is crucial to ensure patient safety.

## 1 INTRODUCTION

Boron neutron capture therapy (BNCT) is a type of radiation therapy that uses charged particles produced by the nuclear reaction of ^10^B and thermal neutrons.^1^ The charged particles produced have a short range and high LET, so if ^10^B is accumulated in tumor cells, they can be selectively destroyed while minimizing damage to normal cells.

The cyclotron‐based epithermal neutron source (C‐BENS) was developed by Sumitomo Heavy Industries in collaboration with Kyoto University as the world's first accelerator‐based neutron source used in clinical trials.^2^, ^3^, ^4^ Following the results of the clinical trial, C‐BENS was approved as a medical device called the NeuCure BNCT System in March 2020 in Japan. In addition, the NeuCure dose engine was developed as the treatment planning software for BNCT with the NeuCure BNCT system. Insurance covered reimbursement for unresectable, locally advanced or locally recurrent head and neck cancer using BNCT has been available since June 2020 in Japan. The system at the Kansai BNCT Medical Center is the same model as the C‐BENS installed at Kyoto University and Southern Tohoku BNCT center.^5^

The neutron energy spectrum of C‐BENS is designed to treat deep‐seated tumors.^6^ In water, epithermal neutrons undergo elastic scattering with hydrogen atoms, losing energy and becoming thermalized. Subsequently, they are absorbed by hydrogen nuclei, leading to attenuation. As a result, the thermal neutron flux peaks at a depth of approximately 20 mm in water.^7^ Therefore, when irradiating the human body, thermal neutrons are also expected to peak at a depth of around 20 mm. For the treatment of malignant tumors located near the skin surface, where epithermal neutrons are not sufficiently moderated, the thermal neutron flux at the skin surface is about 30% of the peak.^2^ It is necessary to shift the thermal neutron flux toward the surface by using a tissue equivalent bolus material.

Boluses are routinely used in X‐ray and electron beam therapy.^8^ For example, in the treatment of cutaneous malignant tumors using electron beams, boluses are used to compensate for the skin surface dose. Similarly, it has been reported that boluses are effective in improving surface dose in BNCT.^9^, ^10^ Moreover, clinical studies targeting malignant melanoma have demonstrated the actual use of boluses in BNCT.^11^ These boluses are considered to be tissue equivalent (i.e., they have a similar electron density to tissue), so during the treatment planning process for photon or electron therapy, the CT number of the bolus is converted to electron density and dose calculation is performed. However, the neutron moderation capability of a bolus is governed by its elemental composition, hydrogen density, and molecular structure. In clinical BNCT treatment planning, material properties (density and elemental composition) are manually assigned. Accordingly, it has been reported that when a bolus is used in a BNCT irradiation field, its neutron moderation capability should be evaluated beforehand.^10^

Since BNCT is neutron‐based therapy, irradiated materials may become radioactive. There are several reports on post‐BNCT activation of patients.^12^, ^13^ Patient activation has been reported to be at a level of less than 200 µSv/h at the irradiated site immediately after irradiation. Post‐irradiation activation of patients has been attributed primarily to the production of nuclides such as ^24^Na and ^38^Cl. However, there are no reports yet on bolus radioactivation caused by neutron irradiation. The detailed elemental composition of the bolus material may not be publicly available. BNCT uses high‐intensity neutron irradiation, and if the composition of the bolus contains elements that would be activated, two problems arise. The first is that the activation of the bolus can result in an unexpected dose to the patient. Second, the activated bolus becomes radioactive waste. Furthermore, the treatment time is relatively long for BNCT, ranging from 30 to 60 min. Therefore, it is necessary to pay attention to the radioactivation of boluses placed within the irradiation field. And if the elemental composition of the bolus is uncertain, the accuracy of the patient dose calculation should be verified.

In this study, we evaluated the tissue equivalence and radioactivation of commercially available boluses using a clinical BNCT irradiation field and its suitability for clinical use.

## 2 METHODS

### 2.1 Evaluation of bolus

**FIGURE 1:**
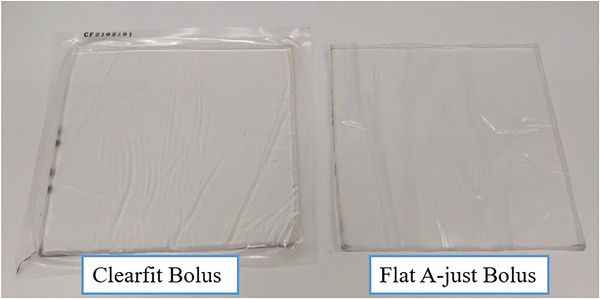
Left) Clearfit Bolus (Bolus A), Right) Flat A‐just Bolus (Bolus B).

Evaluation of two commercially available bolus (Fujidenoro Co., Ltd., Clearfit Bolus and A‐JUST POLYMER Co., Ltd., Flat A‐just Bolus) was conducted (shown in Figure 1). Here, Clearfit bolus was called Bolus A and Flat A‐just bolus was called Bolus B. These boluses are used to improve surface dose in X‐ray therapy and electron beam therapy. Irradiation experiments were performed using the NeuCure BNCT system at Kansai BNCT Medical Center.^14^ A cyclotron accelerator is used to accelerate protons to approximately 30 MeV. When the accelerated protons collide with a beryllium target, fast neutrons are generated. The generated fast neutrons pass through a beam shaping assembly to become thermal neutron beams suitable for BNCT. With a proton current of 1 mA, it is possible to generate a thermal neutron flux of up to 1.4 n/cm^2^/s.

**FIGURE 2:**
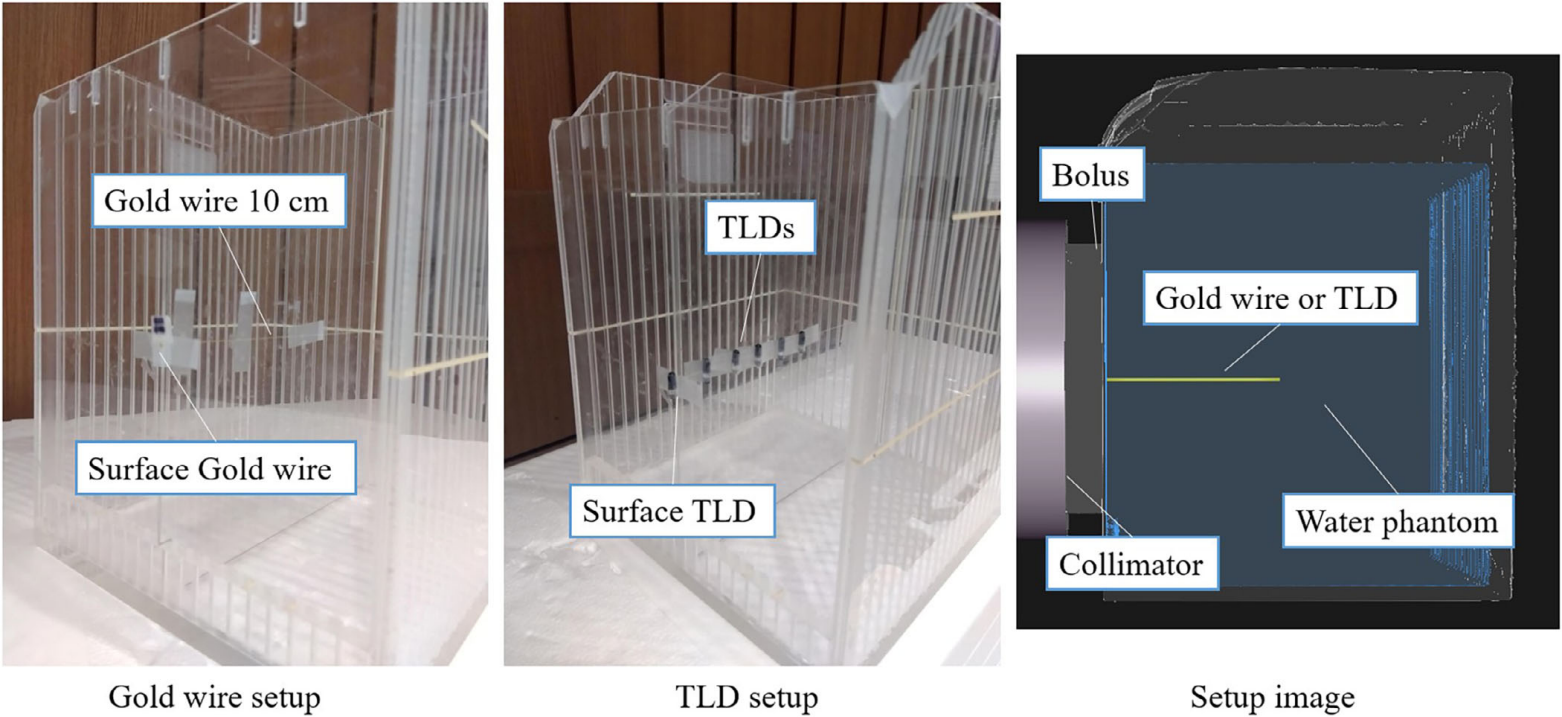
Left) Gold wire setup, Middle) TLD setup, Right) 3D model of setup in TPS.

Bolus A is made of a tissue‐equivalent material and covered with a thin film of polyurethane. The density is 1.02 g/cm^3^. The elemental composition ratio provided by the vendor is H:C:N:O = 10.26:5.27:0.04:1.61. In addition to C, H, O, and N, there are other elements whose details are not known. Bolus B is also made of a tissue‐equivalent polymer gel and covered with a thermoplastic film. Composition ratios and densities are not made available. A 10 and 20 mm thick 150 mm × 150 mm bolus were placed on the surface of a 200 mm × 200 mm × 200 mm cubic water phantom. A 120 mm diameter collimator, which is the reference collimator for this system, was used. The thermal neutron flux distribution in the water phantom was obtained using the gold activation method.^10^ Additionally, the gamma‐ray distribution was obtained using beryllium oxide thermoluminscent dosimeters encased in a special quartz glass (TLDs).^2^ The details of the TLDs used are described elsehwere.^15^ Measurements were taken once at each depth. The measured thermal neutron flux distribution and gamma‐ray distribution were compared with the calculation results obtained with the Monte Carlo‐based treatment planning system (TPS, NeuCure dose engine).^16^ The material composition and physical density of the acrylic phantom was set to PMMA and the water inside the phantom was set to water. The bolus material composition was set to ICRU soft tissue^17^ (H:C:N:O = 0.10:0.11:0.02:0.76) and physical density was set to 1.00 g/cm^3^. Figure 2 shows the setup of the gold wire (The Nilaco Corporation, item number:AU‐171285), TLD, and the geometry modelled in the treatment planning system. Bolus was placed on the surface of the phantom. The bolus was placed so that it was in close contact with the collimator.

### 2.2 Evaluation of bolus radioactivation

The radioactivity of boluses after neutron irradiation was evaluated using an ionization chamber survey meter (Aloka, Lucrest). Measurements were performed on the both surface of the bolus, with and without the beta ray shielding cap. In addition, to identify the activated nuclides in the bolus, gamma ray spectroscopy was performed using an High‐purity Germanium (HP‐Ge) semiconductor detector (ORTEK Inc., GEM20P4‐70) after irradiation. The half‐lives of the activated nuclides were determined by repeatedly short measuring the decay of their characteristic gamma‐ray peaks. The radioactive nuclides were identified based on the estimated half‐life and peak energy values.

## 3 RESULT

### 3.1 Evaluation of bolus

**FIGURE 3:**
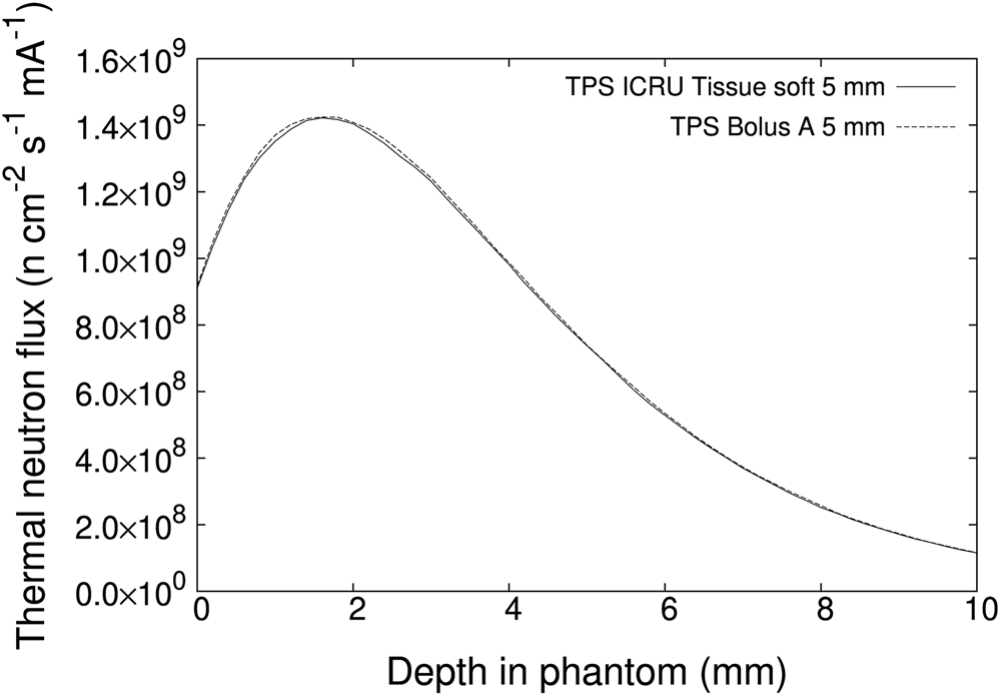
Calculated thermal neutron flux distribution along the central axis in water phantom.

Calculation results with a 5 mm thick bolus are shown in Figure 3. The calculation results using the composition ratio and density of ICRU soft tissue are shown as solid lines, while those based on the composition ratio and density of bolus A are shown as dashed lines.

As can be seen in the figure, the calculation results are almost identical. Therefore, when performing dose calculations, the difference in composition ratio and density between ICRU soft tissue and bolus A can be ignored.

**FIGURE 4:**
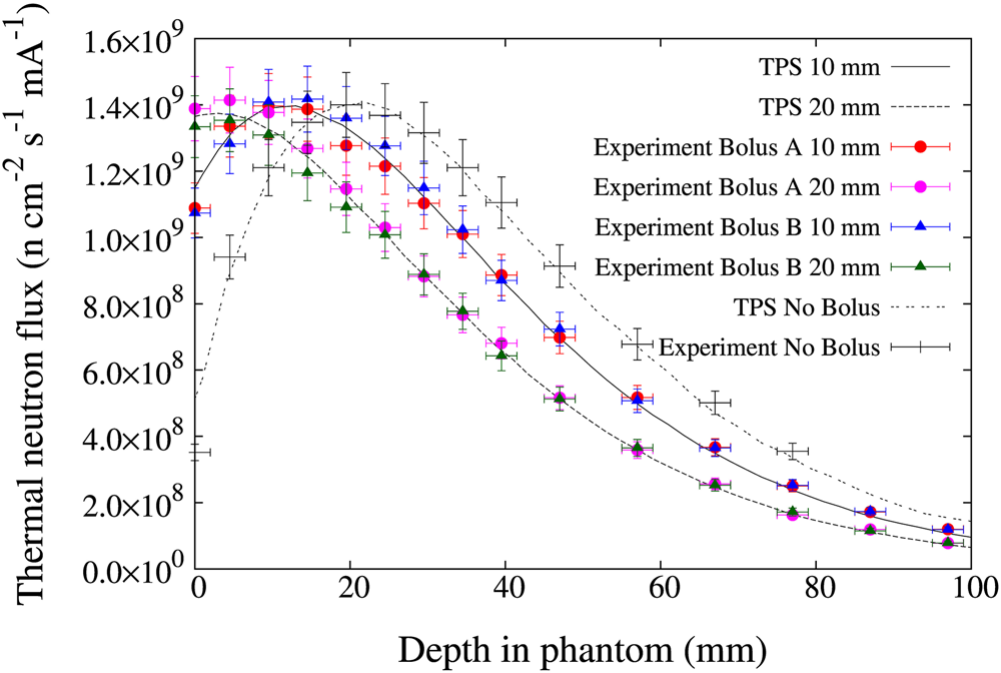
Thermal flux distribution along the central axis in water phantom. Solid circle represents Bolus A and solid triangle represents Bolus B. An error of 7% was applied based on previous reports.^14^, ^18^, ^19^

**FIGURE 5:**
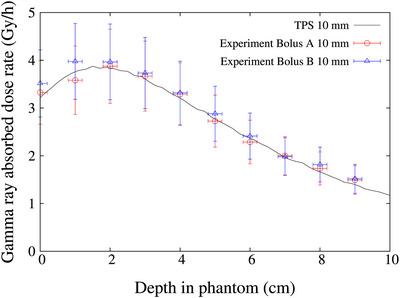
Gamma ray distribution along the central axis in water phantom. An error of 20% was applied based on previous reports.^14^, ^20^

The measured and calculated thermal neutron flux distribution in a water phantom with the bolus placed in front of the phantom is shown in Figure 4. The measured and calculated values showed good agreement within the estimated experimental uncertainties. The error of the experimental values was set at 7% based on previous reports.^14^, ^18^, ^19^ The causes of measurement errors include errors in the mass of the gold wire and errors in the detection efficiency of HP‐Ge. The measured and calculated gamma ray dose rate distributions in the water phantom are shown Figure 5. The measured and calculated values also showed good agreement within the estimated experimental uncertainties. The error of the experimental values for the TLDs were set at 20% based on previous reports.^14^, ^20^

Although the composition ratio of bolus B is unknown, the measurement results and the calculation results assuming ICRU soft tissue show good agreement, so it was possible to use the density and composition of ICRU soft tissue for dose calculations. Therefore, the tissue‐equivalent boluses used in X‐ray therapy and electron beam therapy, which were handled in this study, can be used for dose calculations as ICRU soft tissue.

**FIGURE 6:**
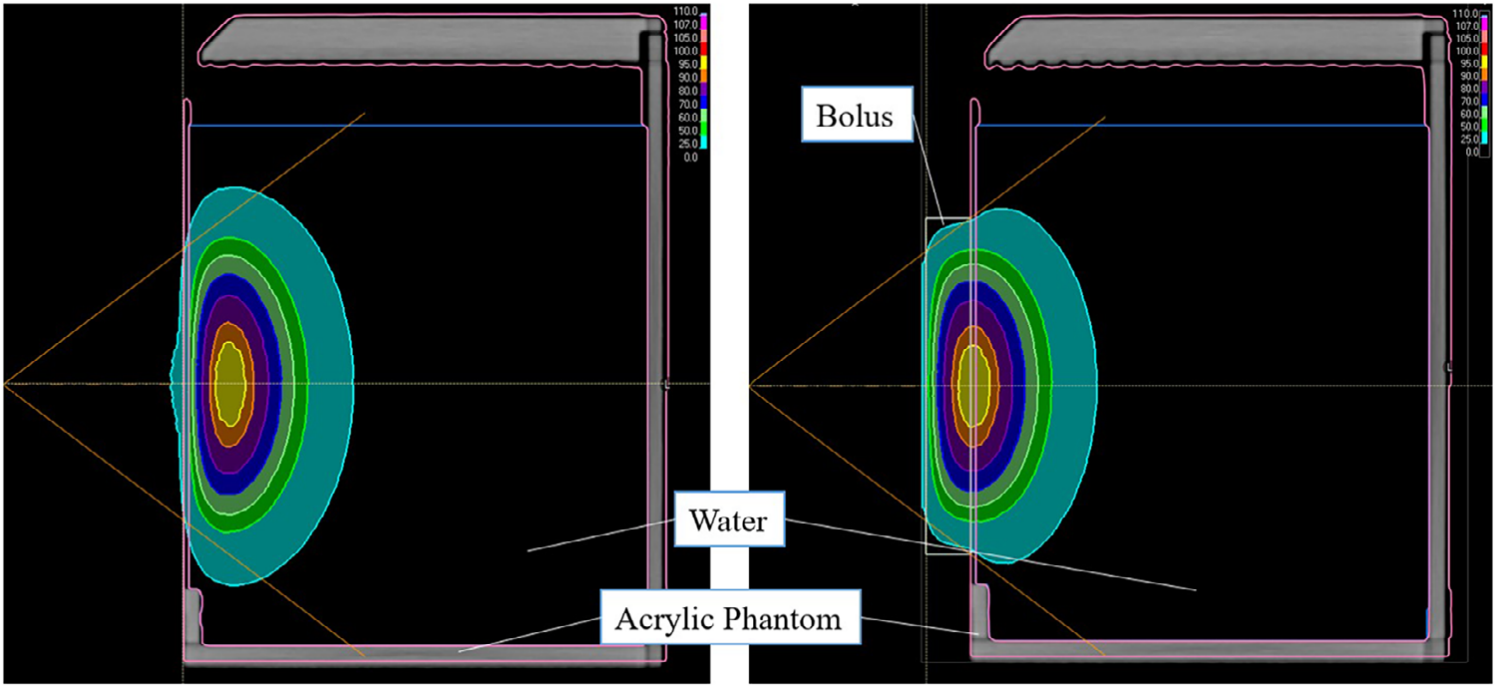
Thermal neutron flux distribution in TPS. Left) Without bolus, Right) With 20 mm thickness bolus.

Figure 6 shows the thermal neutron flux distribution in TPS. By using a 20 mm thick bolus, it can be seen that the peak of the thermal neutron flux shifts to the vicinity of the phantom surface.

### 3.2 Evaluation of bolus activation

**FIGURE 7:**
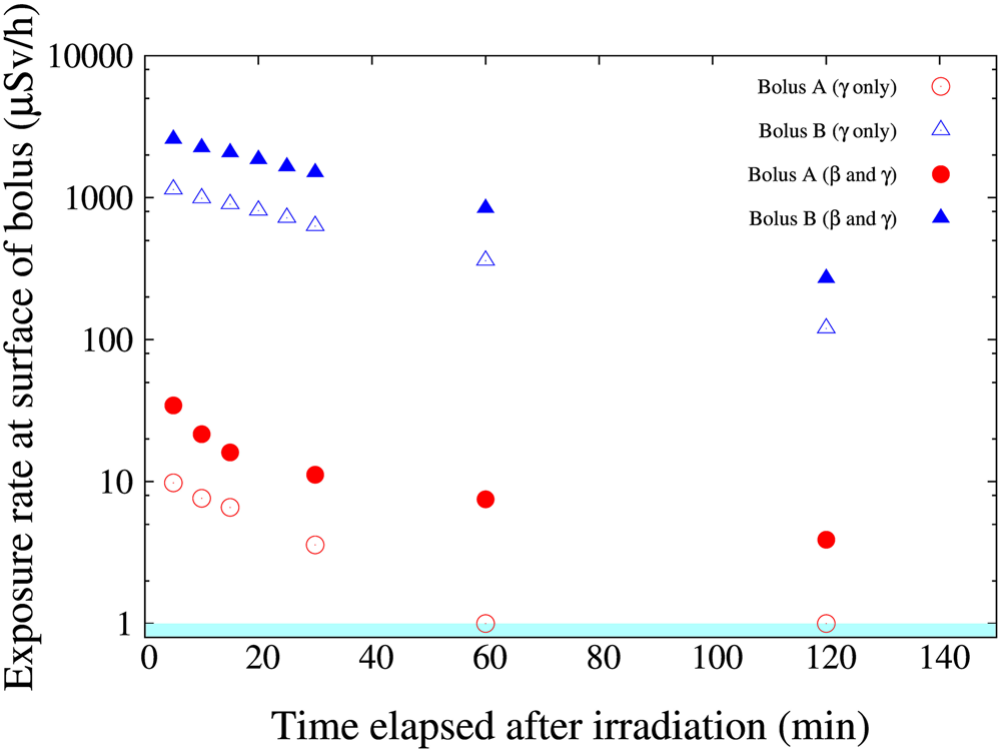
Radioactivity of the different boluses used in this study. Open circle represents radioactivity of bolus A from gamma rays only; solid circle represents both gamma and beta rays. Solid triangle represents radioactivity of bolus B from gamma rays only; solid triangle represents both gamma and beta rays. Below 1 µSv/h is the background.

**FIGURE 8:**
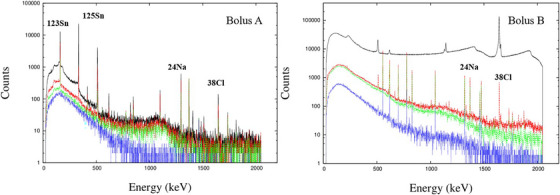
Gamma ray energy spectrum of Left) Bolus A, measured every 2500 s immediately after irradiation. Right) Bolus B. The black line is measured 1 h immediately after irradiation. The red and green lines represent the results of measurements taken 10 h after irradiation ended, starting 5 h after irradiation ended. The blue line represents the results of measurements taken 10 h after irradiation ended, starting 2 days after irradiation ended.

Figure 7 presents the exposure rate measured at the surface of each bolus following a 60‐min irradiation. The data include measurements of gamma rays alone (with cap) and combined gamma and beta radiation (without cap). The dose rates were the same on both surfaces of the bolus. The radioactivity after irradiation varied greatly between the two bolus types. The corresponding major activated nuclides are presented Table 1, and the gamma ray energy spectra presented in Figure 8.

**TABLE 1:** Major activated nuclide of bolus A and bolus B.

Bolus	Nuclide	Peak energy [keV]	Half‐life
A	^123^Sn	160.3	40.1 min
A	^125^Sn	331.9	9.5 min
Both A and B	^38^Cl	1642.6	37.2 min
Both A and B	^24^Na	1368.6	14.9 h

## 4 DISCUSSION

This study assessed the performance of boluses by experimentally measuring and computationally simulating the spatial distributions of thermal neutrons and gamma rays within a water phantom. The NeuCure system typically exhibits a maximum thermal neutron flux at a depth of approximately 20 mm in water. However, when a 10 mm thick bolus was employed, the peak shifted to a depth of approximately 10 mm, indicating that the bolus effectively replaced 10 mm of soft tissue. Furthermore, when a 20 mm thick bolus was employed, the peak shifted to the nearly phantom surface and was equivalent to that of 20 mm of soft tissue. This experimental measurement aligns with the results of treatment planning system calculations that modelled the bolus as a tissue equivalent layer. These findings further corroborate the efficacy of boluses in enhancing surface dose delivery in BNCT.

As boluses are positioned directly on the patient's skin, the radiation emitted from the activated boluses constitutes an additional radiation dose to the patient. The radiation dose from bolus B is estimated to be a maximum of approximately 3 mGy. On the other hand, in BNCT, the skin dose can reach up to a maximum of 15 Gy‐eq in 1 h of irradiation (single fraction dose).^21^ Therefore, the radiation dose from bolus B is sufficiently small compared to the dose from BNCT. However, from the perspective of radiation dose constraints on the lens of the eye (i.e., 100 mSv/5 y, with no single year exceeding 50 mSv), it is a value that cannot be ignored. Considering the high intensity of thermal neutron flux in BNCT, the radiation exposure from activated boluses cannot be overlooked. Consequently, it is imperative to employ boluses that exhibit minimal radioactivation to reduce unnecessary radiation dose to the patient. For this purpose, it is necessary to evaluate not only the thermal neutron and gamma‐ray distributions when a bolus is used, but also the radioactivation of the bolus after neutron irradiation.

On a further note, in X‐ray radiotherapy, dose calculations are usually performed using the electron density of the material. The electron density is obtained from the CT value by means of a CT value‐electron density conversion table. Therefore, materials with equal CT values are assigned as equivalent materials in the treatment planning system, regardless of its chemical composition. Currently, bolus made specifically for BNCT is not commercially available. Therefore, when a bolus is required at a facility that performs BNCT, a bolus that is commercially available for X‐ray or electron beam therapy is typically used (such as the boluses used in this study). These boluses are often considered tissue equivalent, based on the fact the CT number is similar to soft tissue. In BNCT dose calculations, information such as elemental composition and density is specified for each calculation voxel, as the hydrogen density affects the neutron transport.^22^, ^23^ Therefore, it is necessary to evaluate the composition of the bolus before performing dose calculations and utilising it for patients.

## 5 CONCLUSION

An evaluation of commercially available tissue equivalent boluses when used in BNCT irradiation fields was conducted. These boluses are effective for increasing the surface dose, however, due to the radioactivation of the bolus material, a thorough investigation on the material composition of the bolus must be performed before using it for clinical use to minimise unnecessary radiation exposure to the patient.

## AUTHOR CONTRIBUTIONS

Conceptualization and methodology: A.S and N.H. Data analysis and process: A.S, M.N, R.K, A.S. Writing: A.S and N.H. Resources: T.A, S.T, Y.Y. Supervision: H.T, K.N, K.O. All authors have read and agreed to the published version of the manuscript.

## CONFLICT OF INTEREST STATEMENT

The authors declare no conflicts of interest.
